# Pathogenic diversity of RNA variants and RNA variation-associated factors in cancer development

**DOI:** 10.1038/s12276-020-0429-6

**Published:** 2020-04-28

**Authors:** Hee Doo Yang, Suk Woo Nam

**Affiliations:** 1 Department of Pathology, College of Medicine, The Catholic University of Korea, 222 Banpo-daero, Seocho-gu, Seoul, 06591 Republic of Korea; 2 Functional RNomics Research Center, The Catholic University of Korea, 222 Banpo-daero, Seocho-gu, Seoul, 06591 Republic of Korea

**Keywords:** Cancer genomics, Oncogenesis

## Abstract

Recently, with the development of RNA sequencing technologies such as next-generation sequencing (NGS) for RNA, numerous variations of alternatively processed RNAs made by alternative splicing, RNA editing, alternative maturation of microRNA (miRNA), RNA methylation, and alternative polyadenylation have been uncovered. Furthermore, abnormally processed RNAs can cause a variety of diseases, including obesity, diabetes, Alzheimer’s disease, and cancer. Especially in cancer development, aberrant RNAs caused by deregulated RNA modifiers or regulators are related to progression. Accumulating evidence has reported that aberrant RNAs promote carcinogenesis in many cancers, including liver cancer, leukemia, melanoma, lung cancer, breast cancer, and other cancers, in which abnormal RNA processing occurs in normal cells. Therefore, it is necessary to understand the precise roles and mechanisms of disease-related RNA processing in various cancers for the development of therapeutic interventions. In this review, the underlying mechanisms of variations in the RNA life cycle and the biological impacts of RNA variations on carcinogenesis will be discussed, and therapeutic strategies for the treatment of tumor malignancies will be provided. We also discuss emerging roles of RNA regulators in hepatocellular carcinogenesis.

## Cancer: Mixed messages fuel tumor progression

A single gene can generate a variety of RNA products, and changes in this final RNA output can directly contribute to cancer onset and progression. The initial transcription of each DNA sequence yields a raw RNA strand that subsequently undergoes a variety of modification processes that shape its function. Hee Doo Yang and Suk Woo Nam, The Catholic University of Korea, Seoul, have reviewed the potential impact of these mechanisms on malignancy. Some cancers, for example, express RNA sequences that have been inappropriately edited by an enzymatic process called splicing, yielding abnormal RNAs that drive metastasis. Other tumors contain RNAs with atypical chemical modifications, or in which individual nucleotides have been enzymatically converted into other nucleotides. A deeper understanding of these RNA alterations and their impacts could lead to more effective and targeted cancer treatments.

## Introduction

In cancer progression and development, genetic alteration and genomic dysregulation are essential events, but these changes are insufficient for cancer progression. Most cancer cells establish a resistant state via the epigenetic regulation of genes and/or through aberrant alterations regulating physiological conditions in various environments that provide a survival advantage in response to the selective elimination performed by the host^1^.

The advent of high-throughput transcriptome NGS has provided a wealth of information on RNA variation on a genome-wide scale. It has been uncovered by NGS that most human genes have resultant RNA variants, which give a single gene the potential to produce functionally multiple and distinct precursor RNAs (pre-RNAs)^2^. These pre-RNAs made by RNA variations are translated into variant proteins with functional regions omitted because of splicing^3^, or they regulate noncanonical targets^4^. Especially, more altered RNA products can be made in cancer than in normal tissues, and they affect cancer progression.

In this review, RNA variations are categorized into alternative splicing, RNA editing, microRNAs variation, RNA methylation and alternative polyadenylation, all of which yield alternative transcripts. These transcripts affect the functions of protein products or RNA by changing the target molecules, regulating stability, and removing miRNA-mediated translation inhibition or mediating mRNA–protein interactions and protein localization. We also describe the mechanistic functions of RNA variations and focus on cancer progression, especially hepatocellular carcinogenesis. In addition, we discuss ongoing efforts toward targeting-modified RNA and regulating altered mechanisms of RNA variations in cancer therapy.

## Cellular processing to elicit RNA variations

### Alternative splicing

**Fig. 1:**
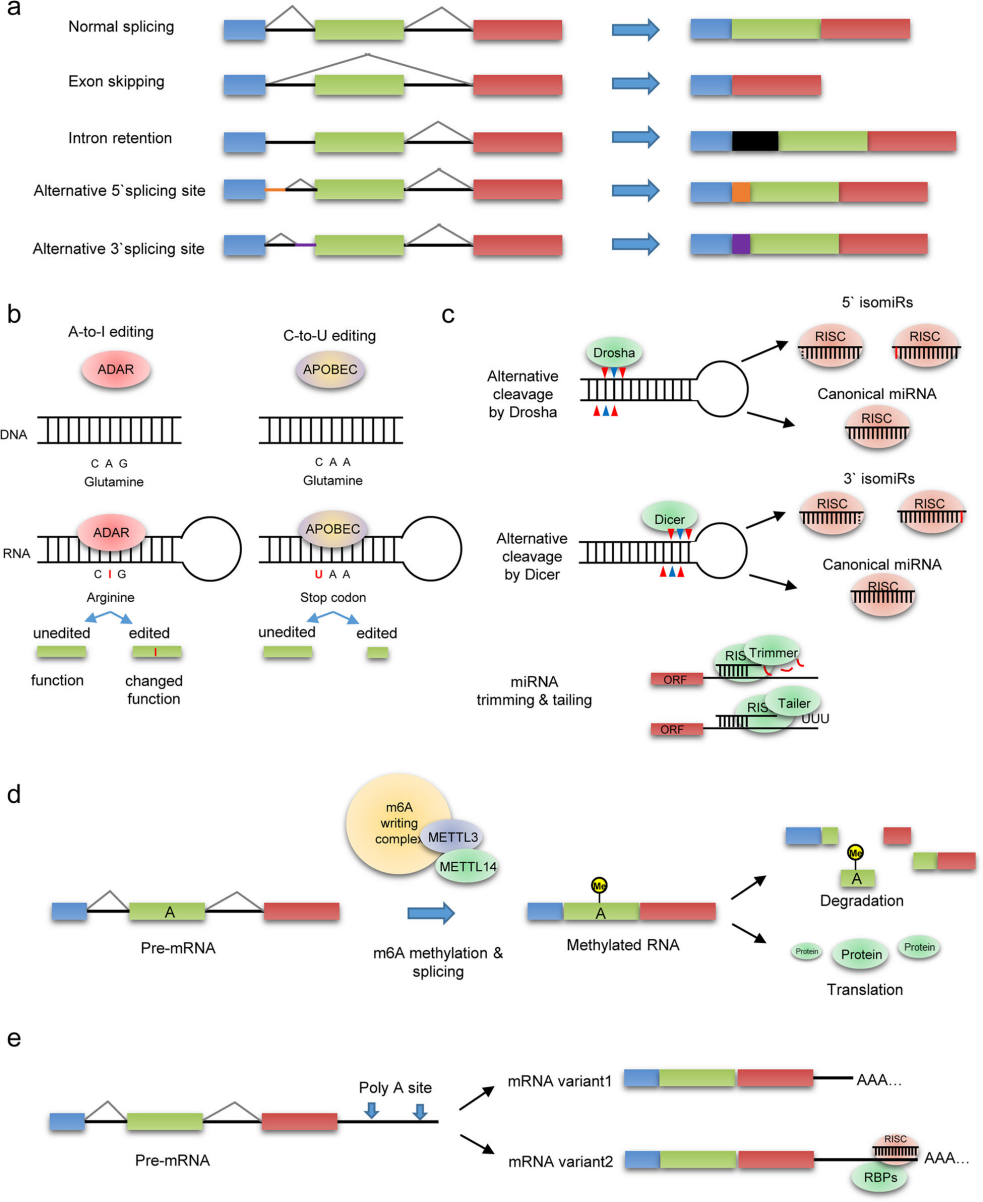
Schematic summary of the diverse processing mechanisms that induce RNA variants. **a** Categorization of alternative splicing (AS) mechanisms. Spicing sites throughout an exon or within introns may disrupt splicing or generate novel aberrant splice sites in the mRNA of a gene. **b** Adenosine and cytidine deaminases are critical RNA editors that play important roles in physiological events. **c** 5′ isomiRs are usually generated by imprecise Drosha and/or Dicer processing, but 3′ isomiRs are mainly produced by postmaturation sequence modifications (trimming and tailing), making 3′ isomiR-trimmed and 3′ isomiR-tailed transcripts, respectively. **d** m6A methyltransferases generate methylated adenosines in RNA and determine the destiny of RNAs. **e** Long 3ʹ untranslated regions (UTRs) contain more regulatory sites that affect the stability and translation rate of the isoforms than the short 3ʹ UTRs made by alternative polyadenylation (APA).

Many more transcripts are produced in the human transcriptome than the number of protein-coding genes. According to the last update of the Encyclopedia of DNA Elements (ENCODE) Consortium, each protein-coding gene has seven different transcript variants in general^5^. These transcript variants are made from alternative splicing of pre-mRNA. Since alternative splicing is a process by which multiple exons are differentially removed from the pre-mRNA transcript, a combination of various exons can be produced, resulting in the production of multiple final mRNA species. Examples of alternative RNA splicing mechanisms include cassette exon inclusion/exclusion, intron retention, mutually exclusive exons, alternative 5′ splice sites, and alternative 3′ splice sites (Fig. 1a)^6^.

In general, gene expression changes of splicing factors may result in alternative splicing. For example, serine/arginine-rich splicing factor 1 (SRSF1, also known as alternative splicing factor 1 (ASF1), pre-mRNA-splicing factor SF2 (SF2) or ASF1/SF2) is frequently overexpressed in various solid cancers with amplification of chromosome 17q23, and it induces alternative splicing^7^. Additionally, mutation of splicing factors may induce alternative splicing. According to Alsafadi and colleagues, mutation of splicing factor 3B subunit 1A (SF3B1) occurs frequently and results in deregulated splicing at a subset of junctions, mostly by the use of alternative 3′ splice sites^8^.

At least three major mechanisms regulating alternative splicing have been reported. First, alternative RNA splicing is regulated by *cis*-elements^6^. These *cis*-elements, such as enhancers of exon/intron splicing, are recognized by serine and arginine proteins (SR proteins) that function as *trans*-acting elements of RNA splicing to promote splice site recognition and exon formation^2^. In contrast, silencers of exon/intron splicing usually relate to *trans*-acting factors such as heterogeneous nuclear ribonucleoproteins (hnRNPs), which suppress splice site recognition and improve exon inclusion^9^.

In addition, single-nucleotide polymorphisms (SNPs) near splice sites can result in RNA splicing alterations. Human genome mutation databases have reported that among disease-causing SNPs, 15% of SNPs are found at splice sites, and over 20% of SNPs are located at splicing enhancers or splicing silencers of exons/introns^10^, suggesting that alternative splicing is a critical step in cancer progression. Another RNA splicing process is connected with transcription initiation and elongation rates^11^. Promoter activities and RNA polymerase II elongation rates influence the recruitment of splicing factors to splice sites in a time-dependent way and therefore regulate the final RNA products^11^. Strongly activated promoters enhance transcription initiation and result in increased recognition of pre-mRNA by the spliceosome. Additionally, the increase in transcription elongation speed after transcription initiation raises the likelihood for the spliceosome to fail at weak splice sites on pre-mRNAs, which would be dependent on the elongation rate.

The third alternative splicing mechanism is related to histone modification. Histone modification near RNA splice sites can affect RNA alternative splicing through recruitment of splicing-regulating proteins and RNA splicing factors. The adaptor protein MORF-related gene 15 (MRG15) interacts with epigenetically modified histone H3 Lys36 trimethylation (H3K36Me3), and hnRNPI (also known as PTB) is recruited to regulate the inclusion of alternatively spliced exons^12^. Additionally, the interaction between H3K36Me3 and the adaptor protein Psip1/Ledgf recruits the splicing factor ASF/SF2, and alternative splicing is promoted^13^. Alternative splicing is regulated not only by the expression/function of RNA splicing factors but also by genomic mutations/SNPs, transcription and elongation rates, and epigenetic modifications. All of these complicated mechanisms contribute to the production of alternatively spliced RNA.

### RNA editing

The unwinding of double stranded RNA (dsRNA) for adenosine to inosine (A-to-I) RNA editing was first found in oocytes of Xenopus laevis^14^. Since then, the study of adenosine deaminases acting on RNA (ADARs) has increased. In mammals, A-to-I conversion is the most common type of RNA editing (Fig. 1b). There are three ADAR genes (ADAR1-3) constituting four isoforms, ADAR1p150, ADAR1p110, ADAR2, and ADAR3. ADAR proteins have three dsRNA binding sites, and homodimerization of the C-terminal regions of ADARs is needed for A-to-I activity^15^. Major RNA editing sites are in noncoding regions, but some sites occur in coding regions and cause amino acid changes^16^. In humans, A-to-I editing in protein-coding genes takes place in introns, resulting in alternative splicing and untranslated regions (UTRs)^17^. Among the UTRs, the A-to-I editing frequency seems to occur more frequently in 3′ UTR sites than in 5′ UTRs^18^. Additionally, Alu repeats, a repetitive short interspersed element (SINE), are the most frequently edited sites. The human genome has a total of 919,035 Alu elements, and RNA editing sites are used in 305,337 Alu elements^19^. In addition, miRNAs are processed by ADARs (ADAR1 and ADAR2) that regulate miRNA biogenesis by A-to-I editing during miRNA maturation^20^.

The activation-induced cytidine deaminase (AID, also known as AICDA)/apolipoprotein B editing complex (APOBEC) family contains evolutionarily conserved cytidine deaminases that have an intrinsic deamination activity for not only C-to-U RNA editing but also for dC (deoxycytidine)-to-dU (deoxyuridine) DNA editing (Fig. 1b). In humans, 11 protein species of the AID/APOBEC family of deaminases are expressed, including AID (AICDA), APOBEC1, APOBEC2, APOBEC3A, APOBEC3B, APOBEC3C, APOBEC3D, APOBEC3F, APOBEC3G, APOBEC3H, and APOBEC4^21^. APOBEC1 site-specifically edits the *APOB* gene and makes premature stop codons, including a short protein isoform of ApoB^22^. Recent reports of transcriptome sequencing showed additional RNA editing sites, mostly in the 3′ UTRs^23^. APOBEC3A, another site-specific C-to-U editing enzyme, showed editing functions after specific stimulation in monocytes and macrophages^24^. In contrast to those edited by ADARs, most targets edited by APOBEC3A are very site-specific and often contain coding regions, resulting in missense/nonsense alterations^25^. Whether other APOBECs, including APOBEC2 APOBEC3C, APOBEC3D, APOBEC3F, APOBEC3G, APOBEC3H, and APOBEC4, also edit cellular RNAs in specific physiological circumstances is unknown^25^. While some APOBEC3 proteins deaminate viral genetic substances or cancer genomes, the cytidine deaminase functions of these enzymes under normal physiologic conditions have not been demonstrated^26^.

### miRNA heterogeneity

With advances in NGS for RNA, it has been suggested that a single miRNA locus can make different miRNA isoforms (isomiRs) that differ in length and/or sequence composition. IsomiRs can be categorized into 5′ isomiRs and 3′ isomiRs, depending on the region of heterogeneity (Fig. 1c). Studies have shown that isomiRs are loaded onto RNA-induced silencing complex (RISC) by binding to Ago1 and Ago2 and function like canonical miRNAs^4^.

Since sequence modifications at the 5′ end of miRNAs after maturation are rare, most 5′ isomiRs are made from imprecise cleavage by either Drosha or Dicer in the processing of miRNA biogenesis. Alternative cleavage by Drosha and/or Dicer frequently arising in miRNA biogenesis has been revealed from NGS for RNA, but it is generally unknown how these variations occur. Some studies have shown that deviations from the expected cleavage site between the basal junction and the apical junction of a pri-miRNA can result in alternative cleavage by Drosha^27^. Furthermore, structural defects affect imprecise Drosha cleavage in the lower stem, altering the overall helical structure of the pri-miRNA, as well as disrupting the interaction between Drosha and the sequence motif of the lower stem^28^. RNA-binding proteins such as hnRNPA1 control Drosha cleavage by changing the secondary structure of the pri-miRNA, which might be one of the reasons why such proteins regulate alternative cleavage by Drosha^29^. Likewise, the cleavage site of Dicer affects the relative position of the pri-miRNA, pre-miRNA loops, structural uncertainty^30^ and Dicer co-factors such as trans-activation responsive RNA-binding protein (TRBP)^31^.

Although alternative selection of cleavage sites by Drosha and Dicer during miRNA biogenesis contributes to the production of 3′ isomiRs, most 3′ isomiRs result from postmaturation modifications that occur almost exclusively at the 3′ end of miRNAs, unlike 5′ isomiRs^32^. These modifications basically include trimming (removal of nucleotides mediated by exonucleases) and tailing (addition of nucleotides mediated by terminal nucleotidyl transferases). Therefore, isomiRs include nontemplated nucleotides; for these reasons, it is difficult to track the origin of isomiRs^33^.

### RNA methylation

N6-methyladenosine (m6A) is one of the common RNA modifications in mammals. m6A RNA methylation plays key roles in the regulation of posttranscriptional gene expression, including effects on mRNAs, miRNAs and long noncoding RNAs (lncRNAs). In addition, m6A methylation regulates various aspects of RNA metabolism, including RNA structure, maturation, stability, splicing, export, translation and decay^34^, and it is catalyzed by an RNA methyltransferase complex (Fig. 1d). Methyltransferase-like 3 (METTL3) and methyltransferase-like 14 (METTL14) are colocalized in nuclear spots and form a stable complex at a stoichiometric ratio of 1:1^35^. METTL3 functions as a catalytic enzyme that binds to S-adenosylmethionine (SAM), while METTL14 plays a structural role in recognizing substrates^36^. Sometimes, the METTL3-METTL14 heterodimer requires adaptor proteins such as Wilms tumor 1-associated protein (WTAP)^35^. Furthermore, WTAP recruits many proteins and lncRNAs, indicating that WTAP functions as a gatherer of other factors to the methyltransferase complex^37^. Additionally, other adaptor proteins, such as KIAA1429^38^, RNA-binding motif protein 15 (RBM15) and RBM15B^39^, have been reported to interact with the METTL3 complex, and downregulation of these adaptor proteins decreases the cellular m6A level. Additionally, METTL16 has been recently discovered as an m6A methyltransferase, which mainly methylates adenosine in 3ʹ UTRs, and its knockdown induced a decrease in m6A of up to 20%^40^. In contrast, fat mass and obesity-associated (FTO) and AlkB homolog 5 (ALKBH5) are the only identified m6A demethylases. Their activity depends on Fe(ii) and α-ketoglutarate, as they use ferrous iron as a co-factor and α-ketoglutarate as a cosubstrate to oxidize the N-methyl group of the m6A site, and as expected, depletion or overexpression of these compounds altered the m6A status^41^.

Similar to DNA methylation, the biological functions of N-methylation in the m6A site are regulated by m6A “readers”^42^. The readers interact with RNAs by two different mechanisms: direct reading or indirect reading. Direct reading means selective binding to the m6A site of RNAs by m6A reader proteins; in contrast, indirect binding implies that the secondary structure of RNAs is changed by m6A modification, which contributes to the accessibility of the sequence by RNA-binding proteins (referred to as the “m6A switch”)^43^. YT521-B homology (YTB) family proteins, including YTHDF1-3^44^ and YTHDC1^45^, can directly bind to m6A-modified RNAs. Additionally, among the nuclear RNA-binding proteins related to pre-RNA processing, hnRNPA2B1 and hnRNPC are responsible for reading the m6A site in a direct and indirect way, respectively. hnRNPA2B1 can directly bind to m6A-modified RNA and regulate miRNA processing^46^. In addition, the presence of m6A modification in RNAs provides hnRNPC with greater access to their U-tract motifs^47^.

### Alternative polyadenylation

For RNAs, 3′ end processing is also critical, similar to other RNA maturation mechanisms. After pre-RNAs are made by RNA polymerase II, the RNA must be processed through 3′ end cleavage and polyadenylation, referred to as simply polyadenylation. One pre-RNA can have multiple polyadenylation sites and generate multiple transcripts that only differ in 3′ terminal sequences, known as alternative polyadenylation (APA) (Fig. 1e). At least 70% of mammalian mRNAs have alternatively polyadenylated isoforms^48^.

Polyadenylation is regulated by *cis*-elements near the polyadenylation site (PAS). In mammals, upstream elements of the PAS include the sequence A[A/U]UAAA, U-rich elements and UGUA elements, and downstream elements consist of U-rich and GU-rich (typically in the form of GUGU) elements^48^. As with other elements, CA sequences, UAUA elements, and G-rich sequences are found near PASs^49^. As mentioned above, alternatively polyadenylated variants are commonly generated, particularly from upstream APA sites^50^. For example, the AAGAAA sequence in upstream APA sites is frequently observed^50^. Additionally, other APA elements, such as upstream UGUA sequences and downstream GU-rich sequences, are found less frequently^50^. These sites have weaker polyadenylation potential and may regulate cell proliferation and differentiation in development or disease processes.

Most APA sites are detected in the 3′ UTR and give rise to mRNA isoforms with different 3′ UTR lengths. 3′ UTR APA influences posttranscriptional regulation in diverse ways. For example, more than half of miRNAs target mRNAs by interacting with UTRs. By shortening the 3′ UTR of mRNAs, miRNA-mediated translation inhibition is broken, resulting in increased translation^51^. In addition, mRNAs can interact with RNA-binding proteins (RBPs) through the 3′ UTR region. In view of this fact, Berkovits and Mayr reported that altered 3′ UTR regions could function as scaffolds interacting with nascent proteins that affect cellular translocation^52^. Although most APA takes place in the 3′ UTR, the sequence upstream of the last exons, usually within introns, is also used to an APA site. Sites of APA upstream of the last exon [termed intronic polyadenylation (IpA) sites when polyadenylated within introns] can alter the translation regions of RNA, similar to making protein variants via alternative splicing. Additionally, proteins without apparent functions can be generated by APA in promoter-proximal introns^53^.

## RNA variations function as cancer drivers

### Alteration of RNA splicing in cancer

**Fig. 2:**
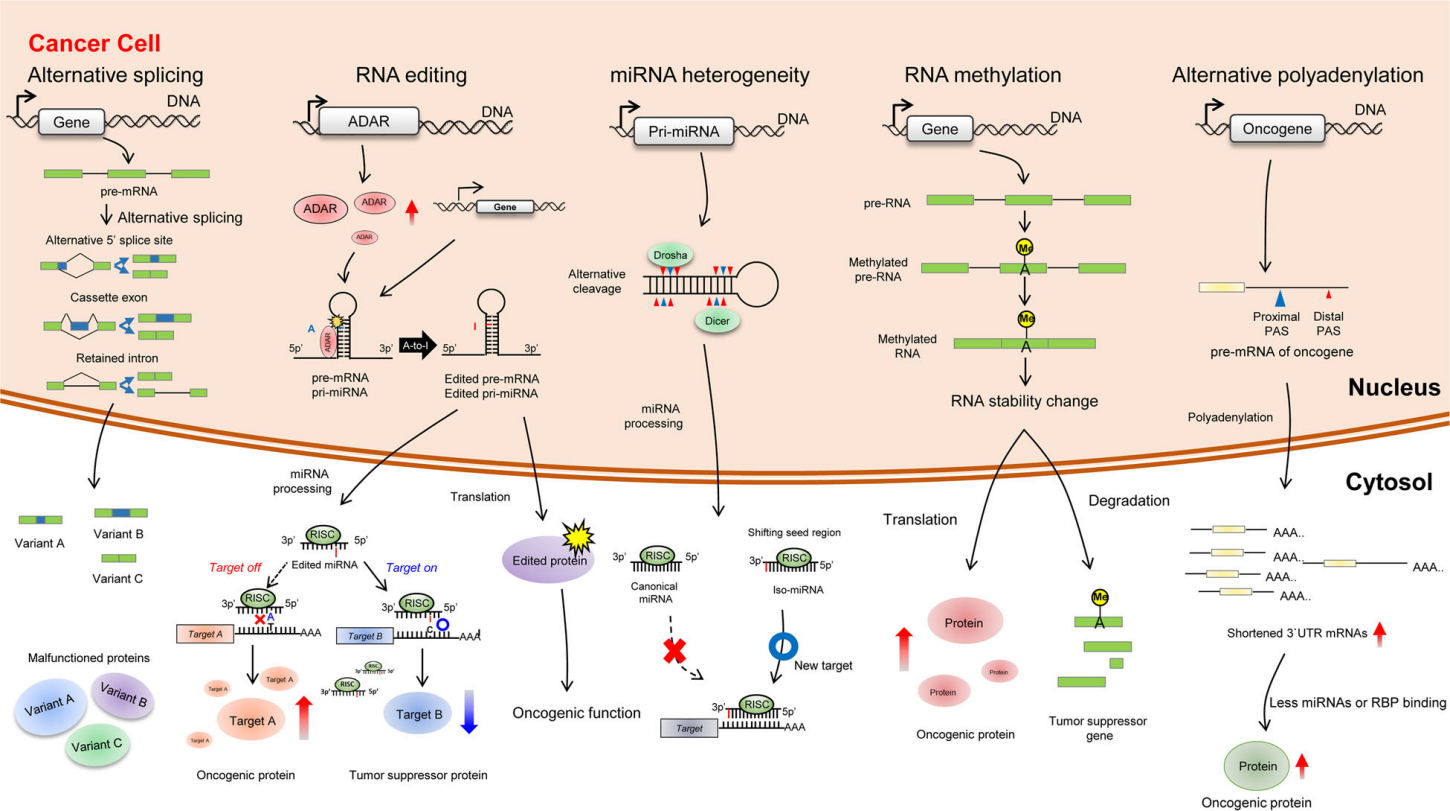
Schematic diagram of RNA variants and RNA variation-associated factors in cancer. The malfunctionated proteins can be made by alternative splicing, which regulates oncogenes or tumor suppressor genes. Also, mRNA or pri-miRNA is edited through indicated RNA processings.

Over the past decade, The Cancer Genome Atlas (TCGA) and the Genotype-Tissue Expression (GTEx) Program have increased the amount of cancer genome and transcriptome data for cancer tissues and normal tissues, respectively (Fig. 2 and Table 1). From these public databases, Kahles and colleagues demonstrated that alternative splicing events in cancer tissues occurred an average of 20% more often than those in normal tissues^54^. These phenomena may be linked to single-nucleotide variants (SNVs) within the genes that break the sequences necessary for canonical splicing. Tumor suppressors, but not oncogenes, are alternatively spliced due to SNV-induced mRNA sequence frameshifts resulting in intron retention (IR)^54^.

**Table 1:** Studies of RNA variations in cancers.

RNA variation types	Cancer types	Refs
Alternative Splicing	Glioma	^56^
	Melanoma	^57^
	Prostate cancer	^58^
	Lymphoma	^59^
	Leukemia	^60^
	Myelodysplastic syndrome	^62^
	Liver cancer	^96^
RNA editing	Ovarian cancer	^63^
	Cervical cancer	^64^
	Myeloma	^65^
	Leukemia	^66^
	Breast cancer	^68^
	Gastric cancer	^69^
	Colorectal cancer	^70^
	Esophageal cancer	^71^
	Melanoma	^74^
	Glioma	^75,76^
	Lung cancer	^77^
	Liver cancer	^97^
MicroRNA variation	Glioma	^80^
	Breast cancer	^82^
	Lung cancer	^83^
RNA methylation	Cervical cancer	^46^
	Kidney cancer	^84^
	Melanoma	^85^
	Lung cancer	^86^
	Breast cancer	^87^
	leukemia	^88^
	Glioma	^89,90^
	Liver cancer	^98,99^
Alternative polyadenylation	Leukemia	^53^
	Sarcoma	^91^
	Lung cancer	^92^
	Breast cancer	^92^
	Colon cancer	^92^
	Myeloma	^93^
	Liver cancer	^100^

Comprehensive analysis of alternative splicing across 32 TCGA cancer types from 8705 patients also showed novel splicing sites caused by mutations^54^. Interestingly, these alternative splicing events occurring in cancer cells elicit disturbances in the expression of certain genes, such as the immune-checkpoint blockade molecules programmed cell death protein 1 (PD-1) and programmed cell death ligand 1 (PD-L1) and T cell-associated genes. The alternatively spliced *PD-L1* gene leads to the production of a truncated and alternatively polyadenylated mRNA and secretion rather than membrane localization and can also negatively regulate T cell function^55^. It was also reported in glioma tissues that some antiangiogenic factors, for example, vascular endothelial growth factor A (VEGFA), can be changed into pro-angiogenic isoforms in carcinogenesis^56^. In melanoma cells, overexpression of alternatively spliced Bcl-2-like protein 1 (BCL2L1, BCLXL) variants confers apoptotic resistance^57^. Furthermore, CD44, which promotes metastasis, has more than 20 alternatively spliced variants and these are common in various cancers^58^. In addition, it has been reported that more than 50% of nucleotide changes can induce splicing changes^59,60^. For instance, according to Supek and colleagues, silent mutations in cancer can affect RNA splicing in oncogenes^61^. As expected, RNAs with long introns are inclined to frequently cause splicing errors and result in the expression of protein-coding isoforms^62^.

### RNA editing in cancer progression

Transcriptome analysis of different types of cancers revealed various forms and levels of RNA editing in cancer cells (Fig. 2 and Table 1). For instance, A-to-I editing patterns are decreased in brain cancer, lung cancer, ovarian cancer, cervical cancer, myeloma and leukemia^63–66^. However, a recent investigation has shown elevated editing levels in noncoding regions, such as intergenic, intronic and 3′ UTR regions, in thyroid, head and neck, breast and lung cancers compared to the levels in corresponding normal tissues, and these increases indicated poor patient survival^67^. These reports indicated potential roles of RNA editing in cancer progression and clinical outcomes.

Editing of the protein-coding region has an effect on protein functions. For example, gamma-aminobutyric acid type A receptor alpha 3 subunit (GABRA3) activates the AKT pathway to promote breast cancer cell migration, invasion and metastasis. However, A-to-I-edited GABRA3 has reduced cell surface expression and suppressed the activation of AKT required for cell migration and invasion in breast cancer metastasis^68^. In addition, RNA editing enzymes, such as ADARs, can play reciprocally oncogenic and tumor suppressive roles through their catalytic deaminase domains. In gastric cancer, ADAR2-induced editing of the podocalyxin-like (PODXL) gene confers a loss-of- function phenotype that neutralizes the tumorigenic ability of unedited *PODXL*^69^. Other RNA editing events in coding regions of the *Ras* homolog family member Q (RHOQ) gene in colorectal cancer^70^ and the solute carrier family 22 member 3 (SLC22A3) gene in esophageal cancer^71^ have been reported to contribute to cancer development.

While the RNA editing sites in coding regions directly alter protein sequences and functions, RNA editing in the 3′ UTR may regulate the binding of miRNAs. The negative correlation between 3′ UTR editing and miR-200 b/c results from repression of mouse double minute 2 homolog (MDM2) by miR-200b/c. Thus, editing in the 3′ UTR of the miRNA binding site of MDM2 may avoid translational repression by miR-200b/c, causing MDM2 overexpression^72^. Given the widespread presence of A-to-I RNA editing sites and microRNA binding sites in the 3′ UTR, RNA editing-regulated miRNA binding might be a common mechanism to control gene expression and may also play a role in physiological and pathophysiological conditions.

Premature forms of miRNAs, being dsRNA molecules, can undergo A-to-I editing at different stages of biogenesis, affecting their maturation and expression. Editing of miRNAs, particularly in the seed region, can significantly alter their target gene, and many miRNAs are aberrantly edited and change their targets in various cancers^73^. In melanoma, reduced editing of miR-455-5p promotes the growth and metastasis of cancer cells by causing it to target the tumor suppressor cytoplasmic polyadenylation element binding protein 1 (CPEB1)^74^. In glioblastoma, modulation of miR-221/222^75^ and miR-376a editing showed antitumor effects by targeting the oncogene RAP2A (a member of the *Ras* family)^76^. Aberrant overexpression of ADAR1 in lung cancer is associated with poor prognosis in patients, as it enhances the editing frequencies of target transcripts such as Nei-like protein 1 (NEIL1) and miR-381^77^. Wang and colleagues systematically characterized the miRNA editing profiles of 8595 samples across 20 cancer types from miRNA sequencing data from TCGA and identified 19 A-to-I RNA editing hot spots^78^. Among these, they demonstrated that edited miR-200b can promote cell invasion and migration through its impaired ability to inhibit *ZEB1/ZEB2* and concomitant ability to repress leukemia inhibitory factor receptor (LIFR), a well-characterized metastasis suppressor^78^.

### miRNA heterogeneity in cancer

Multiple lines of evidence suggest that isomiRs possess unique biological roles. Recent reports indicate that naturally existing isoforms have distinct activities in a wide range of biological processes, including regulation of cytokine expression, facilitation of virus proliferation, promotion of apoptosis and repression of tumor progression. Since 5′ isomiRs usually have a different start position from the defined seed region, 5′ isomiRs may affect target interactions by shifting the seed sequences. This change can allow 5′ isomiRs to regulate noncanonical target genes^79^. A major concern regarding the biological function of 5′ isomiRs is that their expression levels are usually lower than those of their canonical form. Nevertheless, some 5′ isomiRs are expressed enough to function in specific tissues or diseased cells (Fig. 2 and Table 1). For example, in low-grade glioma tissues, a 5′ isomiR of miR-9 is even more expressed than miRNAs with well-known functions, such as let-7, miR-30 or miR-21^80^. Interestingly, miR-9 is produced by three paralog primary miRNAs (pri-miR-9-1, pri-miR-2 and pri-miR-9-3), but the 5′ isomiR of miR-9 is generated only from pri-miR-9-1. The unique structure of pri-miR-9-1 causes altered Drosha cleavage at other positions^80^. Thus, 5′ isomiR creation made by Drosha allows members of pri-miRNA families to achieve functional specialization. Now, this neofunctionalization is of special interest, given that more than 40% of miRNAs are members of a family, and approximately 14% are miRNA paralogs with similar mature sequences^81^. Likewise, recent studies report that 3′ isomiRs function as regulators of miRNA stability beyond intermediates of miRNA degradation. Additionally, the ratio of the 3′ isomiR to the canonical miRNA can be employed to distinguish different grades of cancer^82^. Additionally, it has been reported that many 3′ isomiRs have distinct activities from their canonical miRNAs^83^. However, because 3′ isomiRs, unlike 5′ isomiRs, share their seed region with canonical miRNAs, they may not affect target genes.

### RNA methylation in cancer

N6-methyladenosine (m6A) is the most abundant internal modification in mRNA. Similar to DNA and histone modification, RNA methylation patterns play an important biological function in the regulation of different cellular processes, such as metabolism, embryonic development, and stem cell self-renewal. There have also been links between alterations in m6A levels and abnormal cellular differentiation states present in cancer. m6A modifications have been shown to play a role in leukemia, brain cancer, breast cancer, and lung cancer. m6A modification has been suggested to contribute to cancer progression through alternative pre-mRNA splicing, RNA stability, miRNA processing and lncRNA splicing (Fig. 2 and Table 1)^47,84,85^. Recently, METTL3, an RNA methyltransferase, was demonstrated to promote translation in the cytoplasm in association with ribosomes in lung cancer^86^. METTL3 promotes the growth, survival, and invasion of human lung cancer cells by enhancing mRNA translation through an interaction with the translation initiation machinery. On the other hand, the exposure of breast cancer cells to hypoxic conditions, which is a critical feature of the tumor microenvironment, induces m6A demethylation by ALKBH5 and stabilization of NANOG mRNA, thereby promoting the breast cancer stem cell phenotype^87^. Aberrant m6A modification can affect acute myeloid leukemia (AML). For example, FTO, an m6A eraser, was highly expressed in AMLs with mixed-lineage leukemia (MLL) rearrangements, PML (promyelocytic leukemia)-RARA (retinoic acid receptor alpha) translocations, and/or FLT3 (FMS-like tyrosine kinase-3)-ITD (internal tandem duplication) or NPM1 (nucleophosmin 1) mutations^88^. FTO knockdown also inhibits AML growth, indicating that FTO functions by modulating m6A modification. In glioblastoma, highly expressed ALKBH5, also functioning as an m6A eraser, was associated with a poor prognosis in glioblastoma patients^89^. Knockdown of ALKBH5 reduced the growth of glioblastoma, which could be recovered by catalytically wild-type ALKBH5 but not H204A-mutated ALKBH5^89^. Differentiated glioblastoma stem cell (GSC) cell lines had an increased m6A level, while primary GSC cell lines showed a lower m6A level^90^. Inhibition of METTL3 or METTL14 promoted the growth and self-renewal of GSCs, but overexpression of wild-type METTL3 rather than functionally inactive METTL3 inhibited the growth and self-renewal of GSCs, demonstrating that METTL3 can regulate self-renewal of GSCs through its methyltransferase catalytic function^90^. Overall, m6A modifications and their regulatory proteins play a role in various types of cancers, and therefore, targeting m6A modifications may serve as an effective treatment strategy.

### Alternative polyadenylation in cancer

Alternative polyadenylation (APA) is a molecular process that generates diversity at the 3′ end of transcripts from RNA polymerase II. Recent studies have particularly highlighted the importance of APA dysregulation in cancer (Fig. 2 and Table 1). Deregulation of APA has attracted increasing interest in cancer research because APA generates mRNA 3′ UTR isoforms with potentially different stabilities, subcellular localizations, translation efficiencies, and functions. APA isoforms generated from the same gene may only differ in the length of their 3′ UTRs. Thus, APA may increase the abundance of proto-oncogenes by shortening the 3′ UTR, which effectively eliminates the negative regulatory binding sites. There are several well-known examples of oncogenes with increased levels because of elimination of miRNA recognition sites through APA. For example, in some leukemia patients, upregulation of cyclin D1 is due to truncation of its 3′ UTR, which produces loss of miRNA recognition sites^53^. Similarly, cyclin D2 is also increased by the loss of the miRNA binding site in its 3′ UTR^91^. Notably, recent results also suggest that shortening of the 3′ UTR of an mRNA may allow release of miRNAs to suppress the expression of other mRNAs in *trans*, acting as competing endogenous RNAs (ceRNAs) in lung cancer, breast cancer and colon cancer^92^.

In addition to regulating mRNA expression through APA, 3′ UTRs can also mediate mRNA–protein interactions and protein localization. For instance, APA of short or long isoforms of CD47 mRNA affects the interaction with different protein complexes and thereby guides CD47 localization to either the plasma membrane (long isoform) or the endoplasmic reticulum (short isoform) in breast cancer, cervical cancer, ovarian carcinoma, leukemia, sarcoma, glioblastoma and neuroblastoma^52^. Comprehensive analysis of APA sites and poly A tail lengths in 358 matched tumor-normal samples from seven TCGA datasets suggested that tumor-specific APA events produce the majority of short 3′ UTRs and the subsequent increased gene expression of oncogenes by enabling avoidance of miRNA-mediated gene regulation^93^. Altogether, APA appears to be highly tumor-specific and tissue-specific depending on the cancer type^91,94,95^.

## The pathologic roles of RNA variations in liver cancer

RNA splicing is tightly regulated and closely interacts with genetic and epigenetic machinery. Tumor cells often take advantage of aberrant RNA splicing to develop, grow and progress into cancers. Splicing factor 3B subunit 4 (SF3B4) encodes a core protein in the mammalian SF3b complex, which is part of the U2‐type spliceosome that helps tether the U2 snRNP to the branch site. SF3B4 was overexpressed in a large cohort of HCC patients, and this aberrant overexpression was significantly associated with poor prognosis in HCC patients^96^. Aberrant expression of SF3B4 was demonstrated to modulate the expression of cell cycle and EMT proteins through spliceosome effects on the tumor suppressor kruppel-like factor 4 (KLF4) in hepatocellular carcinoma (HCC).

ADAR-mediated RNA editing is essential for survival in mammals; however, its dysregulation causes aberrant editing of its targets that may lead to cancer. ADAR1 is commonly overexpressed, for instance, in breast, lung, liver and esophageal cancer as well as in chronic myelogenous leukemia, where it promotes cancer progression. In hepatocellular carcinogenesis, antizyme inhibitor 1 (AZIN1) is edited to AZIN1-S367G, a more stable form of AZIN1 with a stronger affinity for antizyme than canonical AZIN1. Antizyme regulates cell growth by binding and degrading growth-promoting proteins such as ornithine decarboxylase (ODC) and cyclin D1 (CCND1), which are essential determinants of the G1/S cell cycle checkpoint. Compared to wild-type AZIN1, edited AZIN1-S367G has stronger antizyme binding and inhibits antizyme-mediated degradation of ODC and cyclin D1, thereby facilitating entry into the cell cycle and increasing the malignancy of liver cancer cells^97^.

m6A is the most abundant and important internal modification of RNA in viruses and eukaryotes. Accumulating evidence suggests that aberrant regulation of m6A turnover is associated with multiple types of cancer, including acute myeloid leukemia, breast cancer, glioblastoma, lung cancer, and liver cancer. The liver is a vital metabolic and digestive organ in pathophysiological processes. Recent studies have suggested that m6A RNA modification highly regulates hepatic function and the development of liver diseases. m6A modification is reduced in HCC tissues compared to normal hepatic tissues^98^. In HCC, METTL14 expression was decreased, and a negative correlation was observed between METTL14 expression and survival in HCC patients. In addition, immunoprecipitation assays showed that METTL14 coprecipitated with DGCR8, recognizing m6A-modified miRNA. miR-126 was decreased and unprocessed pri-miR-126 accumulated in METTL14-depleted cells. It worth noting that METTL14-depleted cells exhibited an enhanced growth rate and metastatic potential, indicating that METTL14 suppressed the malignant properties of HCCs by increasing m6A-dependent miR-126 expression^98^. However, METTL3 is significantly upregulated in HCC, and its overexpression is associated with poor prognosis in patients with HCC. Through m6A sequencing, suppressor of cytokine signaling 2 (SOCS2) was identified to be a target of METTL3-mediated m6A modification. As expected, when the expression of METTL3 was inhibited, SOCS2 mRNA m6A modification was abolished, and its mRNA level was also increased. In addition, m6A-mediated SOCS2 mRNA stability is regulated by YTHDF2, the m6A reader protein, the binding of which shortens the half-life of modified SOCS2 mRNA^99^. These reports suggest the role of m6A-mediated RNA modification in hepatocellular carcinogenesis.

The 3′ UTR alterations induced by APA are regulated by a variety of members of the cleavage and polyadenylation machinery. Nudix hydrolase 21 (NUDT21, also known as CFIm25 or CPSF5), an essential factor for RNA 3′ cleavage and polyadenylation, is involved in APA in HCC^100^. Low expression of NUDT21 is associated with poor prognosis in terms of overall and disease-free survival in patients with HCC. NUDT21 inhibited HCC proliferation, metastasis and tumorigenesis, at least in part, by suppressing proteasome subunit beta type-2 (PSMB2) and CXXC-type zinc finger protein 5 (CXXC5), acting as a tumor suppressor in hepatocellular carcinogenesis.

**Fig. 3:**
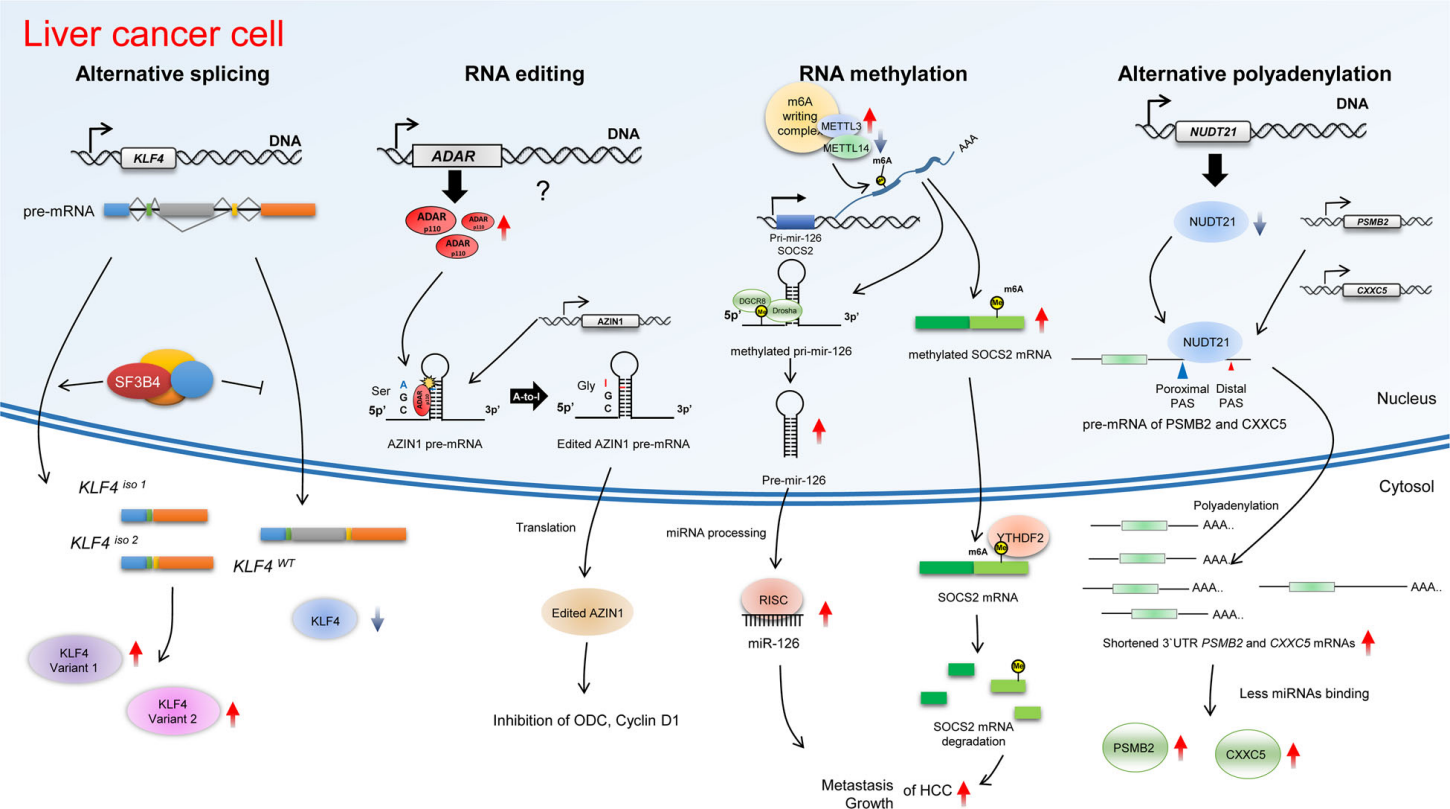
Schematic diagram of RNA variants and RNA variation-associated factors in liver cancer. SF3B4 overexpression triggers aberrant splicing of KLF4 to produce non-functional transcripts. Adenosine-to-inosine editing of AZIN1 transcripts, specifically regulated by ADAR1, leads to a serine to glycine substitution at residue, which causes a conformational alteration.. m6A stimulates miRNA processing by recruiting the Drosha cofactor DGCR8 in the case of the miR-126a, by direct interaction with METTL14. METTL3 epigenetically silences SOCS2 expression through an m6A-YTHDF2-dependent degradation. Downregulation of NUDT21 increases usage of the proximal polyadenylation site in the PSMB2 and CXXC5 3′ UTRs, resulting in marked increase in the expression of PSMB2 and CXXC5 in hepatocellular carcinogenesis.

Overall, various RNA regulating factors may have critical roles in hepatocellular carcinogenesis (Fig. 3).

## Conclusion

Although major developments in our understanding of cancer genomics and molecular biology have been made, the contribution of RNA variations and RNA variation-associated factors to cancer pathogenesis has not been fully elucidated. In this review, we summarized the role of RNA variations and their regulatory factors in diverse aspects of cancer development and progression. Indeed, with advanced technology, as increasingly advanced RNA variants are identified and intracellular functions become known, the pathogenic roles of these RNA variants in cancer development will become more detailed. The identification of functionally important pathologic RNA variants opens up the possibility of therapeutic interventions targeting RNA variants or RNA variation-associated factors in cancers. Thus, regulation of RNA variations could be an effective therapeutic approach for cancer therapy, and a number of approaches have been taken to develop compounds that can experimentally, and sometimes clinically, affect splicing control, resulting in potential novel therapeutics^101^. Therefore, in cancers, changes in the levels of both RNA variants and RNA variation-associated factors are likely to be potential molecular markers for cancer diagnosis and may provide new targets for research and contribute to the development of clinical molecular targeted therapies.
